# The Effect of Poplar *PsnGS1.2* Overexpression on Growth, Secondary Cell Wall, and Fiber Characteristics in Tobacco

**DOI:** 10.3389/fpls.2018.00009

**Published:** 2018-01-19

**Authors:** Tingting Lu, Lulu Liu, Minjing Wei, Yingying Liu, Zianshang Qu, Chuanping Yang, Hairong Wei, Zhigang Wei

**Affiliations:** ^1^State Key Laboratory of Tree Genetics and Breeding, Northeast Forestry University, Harbin, China; ^2^School of Forest Resources and Environmental Science, Michigan Technological University, Houghton, MI, United States

**Keywords:** poplar, *PsnGS1.2*, overexpression, growth, secondary cell wall, fiber, tobacco

## Abstract

The glutamine synthetase (GS1) is a key enzyme that catalyzes the reaction of glutamate and ammonia to produce glutamine in the nitrogen (N) metabolism. Previous studies on *GS1s* in several plant species suggest that overexpression of GS1s can enhance N utilization, accelerate plant vegetative growth, and change wood formation. In this study, we isolated a *GS1* gene, termed *PsnGS1.2*, from *Populus simonii × Populus nigra.* This gene was expressed at a higher level in roots, and relatively lower but detectable levels in xylem, leaves and phloem of *P. simonii × P. nigra*. The protein encoded by *PsnGS1.2* is primarily located in the cytoplasm. Overexpression of *PsnGS1.2* in tobacco led to the increased GS1 activity and IAA content, the augmented N assimilation, and the enlarged leaves with altered anatomical structures. These changes presumably promoted photosynthetic, growth, and biomass productivity. It was noteworthy that the secondary cell walls and fiber characteristics changed remarkably in *PsnGS1.2* transgenic tobacco. These changes aligned well with the altered expression levels of the genes involved in fiber development, secondary cell wall component biosynthesis, IAA biosynthesis, amino acid transport, and starch breakdown. Taken together, the results from our study suggest that catalytic functions of *PsnGS1.2* on N assimilation and metabolism in transgenic tobacco had significant effects on vegetative growth, leaf development, and secondary cell wall formation and properties through acceleration of photosynthesis and IAA biosynthesis, and redirection of carbon flux to synthesis of more cellulose and hemicellulose.

## Introduction

Plants need to absorb inorganic nitrogen (N) in the form of ammonium (NH_4_^+^) or nitrate (NO_3_^-^) from soil to maintain their regular or accelerated growth and development (Jackson et al., 2008). Most NO_3_^-^ molecules are transported to leaves where they are reduced to NH_4_^+^ for incorporation into organic N compounds (Black et al., 2002). The NH_4_^+^ is first assimilated into γ-ketoglutarate to form glutamate (Glu), which is then converted to glutamine (Gln) by incorporating the other NH4^+^ in the presence of Gln Synthetase (GS) (Tobin and Yamaya, 2001). Since Glu and Gln are primary precursors for synthesizing organic N compounds in plants, GS is considered to be a key regulator in the upstream of pathway in which various N compounds are synthesized and mobilized (Miflin and Habash, 2002). There are two types of GS isoforms that have distinct roles in plants, one is the cytoplasmic GS1 type and the other is the choroplastic/plastid GS2 type (Hirel et al., 1984; Dubois et al., 1996; Wang et al., 2015). The GS1 usually assimilates NH_4_^+^ from soil or non-photosynthetic tissues (Ishiyama et al., 2004a), whereas the GS2 mainly acts in the reassimilation of NH_4_^+^ derived from NO_3_^-^ reduction and photorespiration in photosynthetic tissues (Migge et al., 2000; Miflin and Habash, 2002; Betti et al., 2014). Over the past decade, numerous studies have been conducted to analyze the functions of *GS1s* in plant growth and development. The results have indicated that *GS1s* play pivotal roles during N metabolism for vegetative growth, photosynthesis, earlier flowering, and seed development in herbaceous plants (Hirel et al., 1992; Vincent et al., 1997; Fuentes et al., 2001; Oliveira et al., 2002; Man et al., 2005; Martin et al., 2006; Seger et al., 2009). In the woody plants, the *GS1s* have been reported to play principal roles in growth, and development (including wood formation) through assimilation of exterior N and promotion of N recycling processes and closely linked ones (Rennenberg et al., 2010; Coleman et al., 2012). Nevertheless, some studies reported the overexpression of *GS1s* yields no observable alternation of phenotype traits or enhanced N assimilation (Ortega et al., 2001; Suarez et al., 2003). Since not all *GS1s* are regulated in the same manner and located in the same type of cells or organs (Ishiyama et al., 2004b; Martin et al., 2006; Bernard et al., 2008; Castro-Rodriguez et al., 2011), *GS1s* exhibit different functions in N assimilation and metabolism in some transgenic plants (Ishiyama et al., 2004b; Martin et al., 2006; Lothier et al., 2011). These earlier studies suggest that *GS1s* are worth studying and characterizing to benefit humans.

Forest trees grow on infertile land where N deficiency is a critical constraint for their growth and development, it is thus more important and imperative to characterize tree *GS1s* for genetic breeding to enhance N utilization. Poplar is an economically important woody species because of its high growth potential, and variety of uses in industry, such as pulping, paper-making, and biofuel. Although it has been reported that poplar *GS1* family includes up to six genes (Castro-Rodriguez et al., 2011), the well-defined roles of any *GS1s* in poplar have not been identified up to now. In this study, we isolated a full-length cDNA of *PsnGS1.2* from *Populus simonii* × *Populus nigra*, and investigated its functions through overexpression of it in tobacco. Based on previous studies on other *GS1s*, we hypothesized that the poplar *GS1* we isolated may directly and indirectly affect plant performance through accelerating the absorption of N and conversion of inorganic into organic nitrogen, and at same time interacting with plant hormonal balance. To test this hypothesis, we first characterized this *GS1* gene in tobacco. The results explicitly showed that overexpression of the *PsnGS1.2* led to altered N assimilation and metabolism, increased IAA content, accelerated vegetative growth, changed leaf morphology, and altered secondary cell walls and fiber characteristics in transgenic tobacco. Our results suggest that *PsnGS1.2* can be used to create genetically ameliorated plants with significantly augmented biomass production and altered secondary cell walls and fiber characteristics.

## Materials and Methods

### Plant Materials

One-year-old *Populous simonii* × *Populus nigra* trees were propagated and planted in a mixture of turfy peat and sand (2:1 v/v) in the greenhouse. The primary shoot leaves, transition leaves, secondary leaves, primary xylem, transition xylem, secondary xylem, primary phloem, transition phloem, secondary phloem and roots were collected and immediately frozen in liquid Nitrogen and stored at -80°C. The RNA was isolated according to a previously published method (Liao et al., 2004) and later treated with DNase I (Qiagen) to remove genomic DNA (Kolosova et al., 2004).

### Cloning *PsnGS1.2* from *P. simonii × P. nigra*

Five microgram total RNA were used for the synthesizing cDNAs using SuperScript II Reverse Transcriptase (Invitrogen). The full *PsnGS1.2* cDNA was amplified from *P. simonii × P. nigra* with gene-specific primers (Supplementary Table S1). The PCR product was cloned into pMD18-T vector (TaKaRa), and then transformed into *Escherichia coli* cells (DH5α) for validation by Sanger sequencing.

### Sequence Comparisons and Phylogenetic Analysis

BLASTX and BLASTP^1^ were used to analyze the sequence similarity of the cDNA and deduced protein of *GS1s*, respectively. The conserved domains of GS1s were searched by CDD algorithms^1^. Multiple sequence alignment was carried on using ClustalW2^2^ with default setting. *PsnGS1.2* homologous gene sequences from *Chlamydomonas reinhardtii* (*CrGS1.2*-30784960), *Zea mays* (*ZmGS1.2a*-30991251), *Arabidopsis thaliana* (*AtGS1.2a*-19668213), *Oryza sativa* (*OsGS1.2*-33138580), *Morchella esculenta* (*MeGS1.2*c-32327725), *Populus trichocarpa* (*PtrGS1.2a*-27031680 and *PtrGS1.2b*-27016370), *Eucalyptus globulus* (*EgGS1.2*-32071611), and *Salix purpurea* (*SpGS1.2*-31427432) were retrieved from Phytozome Database^3^ using TBLASTN with the PsnGS1.2 sequence being used as the query. A total of 70 putative GS1 protein sequences from 44 plant species (Supplementary File) were aligned using ClustalW2, and the resulting sequences were used to construct a phylogenetic tree by the neighbor-joining method in MEGA 5.0 software, with 1,000 replicates for bootstrap analysis, and a 50% cutoff value.

### Subcellular Localization

The full-length coding region of *PsnGS1.2* without termination codon was amplified using specific primers (Supplementary Table S2) and then fused to the N–terminal of GFP driven by CaMV 35S promoter in pGWB5 vector. The 35S-PsnGS1.2-GFP fusion construct was delivered into onion epidermal cells via particle bombardment (GJ-1000). The GFP fluorescent images were photographed with confocal microscopy (Leica TCS SP5) at 24 h after bombardment.

### Transformation of *Nicotiana tabacum*

The *PsnGS1.2* was amplified with specific primers (Supplementary Table S3), and then inserted into the pROKII vector at the position immediately downstream of CaMV 35S promoter. The pROKII-*PsnGS1.2* was first transferred into *Agrobacterium tumefaciens* EHA105 using the freeze-thaw method. Tobacco plants (*Nicotiana tabacum*) were then transformed as described previously (Maiti et al., 1993). Transgenic tobacco lines were selected on MS medium containing 250 μg/ml kanamycin and 500 μg/ml carbenicillin. The T1 seeds from self-pollinated plants were germinated on MS medium with kanamycin (25 mg/L) to produce T1 generation transgenic lines. We repeated this process to obtain the T2 generation seeds. The genomic DNA of T2 seedlings was amplified by regular PCR using the PROKII sequencing primers listed in Supplementary Table S3 to verify whether *PsnGS1.2* was integrated into tobacco genome. All tested *PsnGS1.2* transgenic lines and wild-type (WT) were grown in the greenhouse and subsequently used for characterization.

The plastichron index (PI) method was used to determine tobacco growth phases. The first leaf larger than 5 cm was named PI0. Then the leaf immediately below PI0 was defined as PI1. Stem segments between PI5 and PI8 were used for breaking force, secondary cell wall thickness, cell wall chemical composition, and gene expression analyses. Leaves of PI3, PI4, and PI5 were measured for leaf lengths and widths, *PsnGS1.2* transcript abundance, chlorophyll contents, biochemical and physiological parameters. Leaf samples for biochemical analyses were obtained from central regions of leaves by using a cork borer (4-mm diameter). Disks prepared from each transgenic line or WT were thoroughly mixed. Samples were weighed, frozen in liquid nitrogen and stored at -80°C.

### Gene Expression Analysis

Five microgram total RNA from multiple tissues of *P. simonii* × *P. nigra* and tobacco plants were used for synthesing cDNA, respectively. Samples of cDNA were run in triplicate with the SYBR premix ExTaq kit (TaKaRa) and an Applied Biosystems 7500 Real-Time PCR System to determine the critical threshold (Ct). The *PsnGS1.2* expression levels in poplar were detected by the real-time quantitative PCR (qRT-PCR), and the primers used for qRT-PCR of *PsnGS1.2* and reference gene, *PsnACTIN1*, are listed in Supplementary Table S4.

The expression levels of *PsnGS1.2* in tobacco lines were determined by reverse transcription (RT)-PCR using *NtACTIN2* as an internal reference. All the primers used are shown in Supplementary Table S4.

Analysis of expression levels of genes involved in cell expansion and elongation (*ExpansinA, ExpansinB, TIP1;3, TIP1;4, XTH5*, and *XTH8*) (Aspeborg et al., 2005; Hacke et al., 2010), programmed cell death (*XSP1, XCP2, SCPL45*, and *SCPL49*) (Plavcova et al., 2013), and cellulose (*CesA4, CesA7*, and *CesA8*) (Appenzeller et al., 2004), hemicellulose (*FRA8, IRX9*, and *IRX10*) (Wu et al., 2010) and lignin biosynthesis gens (*PAL1, PAL4, CAD14, CAD19, 4CL1, 4CL2, HCT*, and *CCoAOMT1*) (Raes et al., 2003), IAA biosynthesis gene *(ASA1*) (Stepanova et al., 2005, 2008), membrane amino acid transporter gene (*AAP11*) (Couturier et al., 2010), and the β-amylase gene (*AMY1*) (Castro-Rodriguez et al., 2016) in tobacco were performed using gene-specific primers (Supplementary Table S5). The primers of *NtACTIN2*, used as an internal control, were listed in Supplementary Table S4. Quantification of gene expression relative to *PsnACTIN1* or *NtACTIN2* was calculated using the delta-delta CT method (Livak and Schmittgen, 2001).

### Phonotype Trait Measurement

The heights and stem diameters (3 cm above the root collar) were measured for each plant. The fresh weights were determined immediately after the whole plants were harvested. Then, the materials were placed in an oven, heated for 10 min at 100°C, and then heated at 75°C until the weights did not change. The final unchanged weights were recorded as dry weights.

### Biochemical Analysis

For assessment of total GS enzyme activities, the samples were respectively ground on ice with extraction buffer consisting of 70 mM MOPS (pH 6.8), 10 mM MgSO_4_, 2 mM dithiothreitol, 5 mM glutamate, 0.1% (v/v) Triton X-100 and 10% (v/v) ethanediol. The final pH of extraction solution was 7.5–8.5. The homogenates were centrifuged at 4°C, 12,000 *g* for 30 min, the supernatant was analyzed for total GS activities. Total GS activities were measured in a preincubated assay buffer (37°C) consisting of 70 mM MOPS (pH 7.6), 100 mM glutamate, 50 mM MgSO_4_, 15 mM NH_2_OH, and 15 mM ATP. The reaction was terminated after 30 min at 37°C by adding acidic FeCl_3_ solution (370 mM FeCl_3_, 670 mM HCl, 200 mM trichloroacetic acid) (Lynch and Barbano, 1999). The reaction was set for 5 min to allow colors to develop, and then centrifuged at 4,000 *g* at room temperature for 10 min, and the supernatant was transferred into a new tube. The amount of formed γ-glutamyl hydroxamate (γ-GHA) was determined with spectrophotometer at 540 nm by reference to the standard curve of γ-GHA.

For soluble protein analysis, each sample was homogenized by grinding on ice with extraction buffer [10 mM Trizma (pH 7.5), 5 mM sodium glutamate, 10 mM MgSO_4_, 1 mM dithiothreitol, 10% (v/v) glycerol, and 0.05% (v/v) Triton X-100, pH 8.0]. The homogenates were then centrifuged at 4°C, 12,000 *g* for 20 min (O’Neal and Joy, 1973; Bradford, 1976). The soluble protein concentration of the supernatant was measured by the Bradford protein assay using BCA protein assay kit (Pierce Biotechnology, United States) (Minocha and Long, 2004). Bovine serum albumin was used as the standard protein.

For free amino acid contents analysis, the freshly harvested samples (∼200 mg) were placed in Eppendorf tubes with 800 μl 5% (v/v) ice-cold perchloric acid, frozen at 20°C and then thawed. This process is repeated for three times, and then centrifuged at 13,500 *g* for 10 min. Amino acids were analyzed by HPLC according to the method of Minocha and Long (Minocha and Long, 2004). External standards consisted of a mix of 23 amino acids.

The total N contents were determined using Kjeldahl method (Lynch and Barbano, 1999). The free NH_4_^+^ was extracted from fresh materials with 60% (v:v) methanol and determined by the salicylate dichloro-isocyanurate assay (Berthelot reaction) (Husted et al., 2000), whereas the free NO_3_^-^ was extracted from the fresh materials with hot deionized water (80°C) and the supernatant was determined by salicylic acid-H_2_SO_4_ method using KNO_3_ as the standard (Cataldo et al., 2008).

Finally, the Free IAA contents were measured using GS-MS according to the procedure of Muller as described (Pollmann et al., 2009).

### Determination of Break Forces

The breaking forces, which has been reported to be correlated with the cellulose content in stem of maize (Dhugga, 2007), refers to the tensile or bending strength used to break stem. The breaking forces of stem segments were analyzed using YYD-1 plant stalk analyzer according to the manufacturer’s instructions (Zhejiang Top Instrument Co., Ltd.).

### Scanning Electron Microscopy

Stem segments were prepared by freeze-drying for scanning electron microscopy (SEM) (S-4800, HITACHI). Dry segments were mounted on aluminum stubs using carbon tape with conductive silver paint applied to the sides to reduce sample charging. The segments were then sputter-coated with gold in an E-100 ion sputter. Imaging was performed at beam accelerating voltages from 12.5 to 25 kV. The secondary wall thicknesses of fibers in the SEM micrographs were quantified in a randomly selected area of 45 cells using Image J software^4^.

### Determination of Chlorophyll Contents and Photosynthetic Parameters

To determine chlorophyll contents, leaves were randomly selected and measured for total chlorophyll contents following described previously (Qiu et al., 2013). The photosynthetic rate, stomatal conductance, and transpiration rate were acquired using Li-6400XT portable photosynthesis system (Li-COR) according to the manufacturer’s instructions.

### Histological Analysis

Stem sections (1 μm thick) cut with Leica EM UC6 microtome were stained with 0.01% Calcofluor White, and the cellulose was observed with an inverted UV fluorescence microscope. Under this condition, only secondary walls exhibited brilliant fluorescence. At the same time, some stem sections (50 μm thick) were stained with phloroglucinol-HCl for observing lignin, which takes on bright red color under a light microscope. To examine the xylan, 1-μm-thick sections were probed with LM10 monoclonal antibodies, which are capable of binding to 4-*O*-methylglucuronoxylan (McCartney et al., 2005), and detected with fluorescein isothiocyanate-conjugated secondary antibodies. The fluorescence-labeled xylan signals were visualized and imaged with an Olympus DX51 light microscope.

### Determination of Contents of Cellulose, Hemicellulose, and Lignin

The determination of the contents of lignin, cellulose, and hemicellulose was conducted with the ANKOM 2000i Automatic fiber analyzer (Ankom). The procedures are recapitulated as follows.

First, the stems of tobacco were placed in an oven, heated for 10 min at 100°C, and then heated at 75°C until the weights did not change. Then, the dried stems were ground to powders. 0.5 g material of each sample was placed in a filter-bag and soaked in acetone for 10–20 min for degreasing. This step was repeated once before the material in the bag was dried for 10 min. After adding 1L neutral clean solution (30 g sodium dodecyl sulfate, 18.61 g ethylene diamine tetraacetic acid disodium salt, 6.81 g sodium tetraborate, 4.56 g disodium hydrogen phosphate, 10 ml triethylene glycol, adding 1 L distillation water and mixing, pH 6.9–7.1) and 20 g sodium sulfite in the material, the neutral detergent fiber (NDF) procedure of ANKOM 2000i Automatic Fiber Analyzer (Ankom) was run for 110 min. Then the material in each filter-bag was taken out and placed in an oven, heated for 3 h at 102°C. The contents of NDF, including hemicellulose, cellulose and lignin, were subjected to gravimetric analysis.

After the NDF content was determined, the material in each filter-bag was soaked in 1 L acid solution (20 g cetyltrimethyl ammonium bromide, 1 L 1 N sulphuric acid solution), the acid detergent fiber (ADF) measuring procedure was performed with ANKOM 2000i Automatic Fiber Analyzer. At end of the procedure, the material in each filter-bag was taken out and placed in an oven, heated for 2∼4 h at 102°C. The content of ADF, including cellulose and lignin, was subjected to gravimetric analysis. The hemicellulose content was the NDF content minus ADF content.

After the ADF content was determined, the material in each filter-bag was soaked in 72% (v/v) sulphuric acid solution and set aside for 3 h. The filter-bag was washed with water for 5∼10 times until pH is neutral. The material in each bag was placed in an oven and heated for 4 h at 102°C. Then, each material bag was placed in muffle furnace and carbonized for 30 min at 300°C without covering the lid, and then heated to 600°C. The rest material, acid detergent lignin (ADL), was subjected to gravimetric analysis. The content of cellulose was the content of ADF minus ADL content.

### Fiber Length and Width Analysis

Stem segments with approximate dimension of 2 mm × 2 mm × 30 mm were harvested and immersed into Franklin solution (1:1 peroxide and glacial acetic acid) with 3.6% (g/v) sodium hypochlorite for 20 h at 70°C. Upon decanting the solution, the materials were immersed in pure Franklin solution for 4 days at 70°C, washed in a vacuum with deionized water until the materials reached a neutral pH, dried for 24 h at 105°C, and then re-suspended in 10 ml of deionized water. The fiber lengths and widths were obtained by counting 25–40 fibers per second on Fiber Quality Analyzer (FQA).

### Statistical Analysis

The student’s *t-*test^5^ was used to statistical analysis. In the figures, mean values and standard deviation (SD) of three biological replicates are shown. Difference between two groups of data for comparisons in this study were evaluated by statistical significance (^∗^*P* < 0.05) or extreme significance (^∗∗^*P* < 0.01).

## Results

### Clone and Characterization of the *PsnGS1.2* from *P. simonii × P. nigra*

**FIGURE 1:**
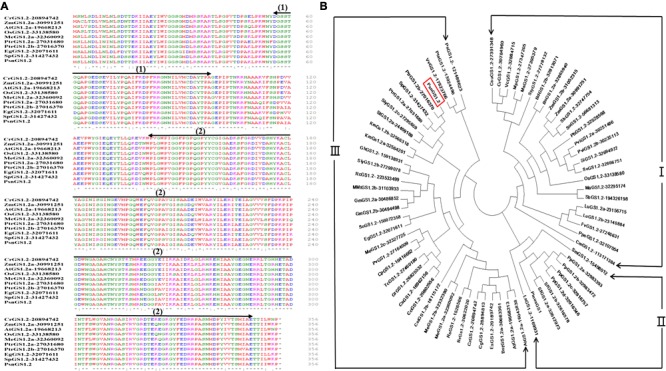
Comparison of the amino acid sequences and phylogenetic relationships among GS1.2 proteins including PsnGS1.2. **(A)** Comparison of the amino acid sequences among nine GS1.2 proteins including PsnGS1.2, Arrows indicate conserved domains: (1) Glutamine synthetase, beta-Grasp domain (56–97 aa), and (2) Glutamine synthetase, catalytic domain (141–348 aa). Residues are colored according to their polarity properties (neutral non-polar as black, neutral polar as green, acidic as red, and basic as blue). **(B)** Phylogenetic analysis of 72 GS1.2 proteins from 44 species and the protein sequences of these genes are enclosed in the Supplementary File 1. PsnGS1.2 is shown in a red rectangular frame. The protein sequences and gene IDs were downloaded from the Phytozome database (https://phytozome.jgi.doe.gov/pz/portal.html).

We obtained a *PsnGS1.2* cDNA of 1071 bp length from *P. simonii × P. nigra*. The protein sequence of *PsnGS1.2* is 98% identical to the protein encoded by *Potri.007G069600.1* in *Populous trichocarpa*, while cDNA sequence is 93% identity to *Potri.007G069600.1*. The deduced protein sequence comparisons demonstrated that the GS1 proteins are highly conserved in different species (**Figure 1A**). The conserved domain analysis revealed that PsnGS1.2 contained two conserved domains: Glutamine synthetase, beta-Grasp domain (IPR008147) and Glutamine synthetase, catalytic domain (IPR008146) (**Figure 1A**), which are typical structure characteristics of GS proteins (Zhu et al., 2014). These results showed that the PsnGS1.2 belongs to the GS1 subfamily.

Phylogenetic analysis of PsnGS1.2 together with 70 GS1.2 protein sequences from 44 species, including green alga, microbes, and higher plants revealed the existence of three large phylogenetic classes of GS1.2 proteins in these species (**Figure 1B**). PsnGS1.2 was found in the Clade III, the largest one with 4 sub-clades that comprise of 37 GS1.2 proteins from 32 species. The Clade I and II comprised 25 and 9 GS1.2 proteins from 22 and 5 species, respectively. It was also remarkable that the GS1.2s of the same species did not show up in the same sub-clades or classes. For example, SlyGS1.2-27278132, SlyGS1.2b-27299078, and SlyGS1.2c-27295659 from *Solanum lycopersicum*. In addition, the GS1.2s from monocotyledon, dicotyledon, herbage, woody plants, and algae species belong to the same sub-clades, indicating that the evolution of GS1.2s is unparalleled with that of species. Moreover, the PsnGS1.2 is not clustered into the same clade with well-characterized Pinus GS1 (**Figure 1B**), which means that the functions of *PsnGS1.2* may not be inferable from other known functional *GS1s* of woody species.

### Tissue-Specific Expression and Subcellular Location of PsnGS1.2

**FIGURE 2:**
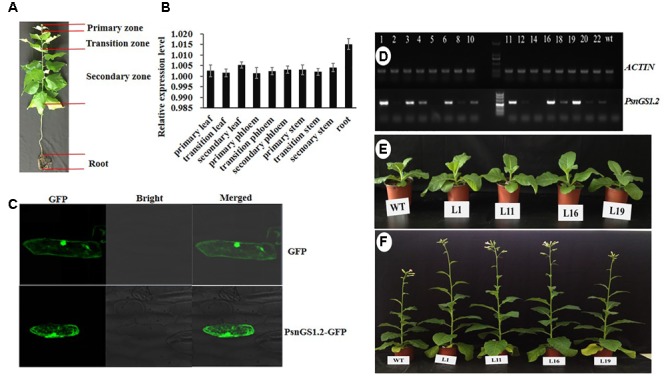
The expression patterns of *PsnGS1.2* and morphologies of *PsnGS1.2* transgenic tobacco and wild-type. **(A)** The illustration of different tissues in 1-year-old *Populus simonii × Populus nigra*. **(B)** Quantitative RT-PCR analysis of relative expression levels of *PsnGS1.2* in ten tissues of 1-year-old *P. simonii × P. nigra*. The *PsnACTIN2* was used as an internal control. Error bars represent the standard deviation (SD) of three biological replicates. **(C)** Subcellular location of PsnGS1.2 proteins in onion epidermal cells. **(D)** RT-PCR analysis of *PsnGS1.2* expression levels in transgenic lines. The numbers from 1 to 22 denote different *PsnGS1.2* transgenic lines, WT refers to wild-type tobacco. *NtACTIN2* was used as an internal control. Morphologies of 1-month old and 3-month old *PsnGS1.2* transgenic lines **(E)** and WT **(F)**, respectively.

*PsnGS1.2* expression patterns in ten tissues of *P. simonii* × *P. nigra* were analyzed by quantitative RT-PCR (**Figure 2A**). The results indicated that the expression level of *PsnGS1.2* in the roots was the highest among all examined tissues (**Figure 2B**), indicating that it mainly functions in N assimilation. In addition, *PsnGS1.2* was also expressed in the primary, transition, and the secondary stems, suggesting that *PsnGS1.2* can also function in growth and wood formation.

To verify whether PsnGS1.2 belongs to the category of cytoplasmic GS1s, an *in vivo* localization experiment was performed by transient overexpression *PsnGS1.2* in onion epidermal cells through particle bombardment. As shown in the **Figure 2C**, the GFP proteins and PsnGS1.2-GFP fusion proteins were both detected primarily in cytosol. Such a result indicated that the *PsnGS1.2* encodes a cytosol-localized protein.

### Alternation of Growth-Related Traits in *PsnGS1.2* Transgenic Tobacco

**FIGURE 3:**
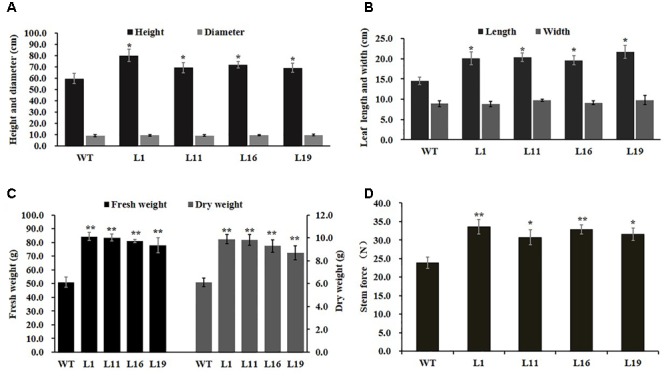
Developmental and growth of *PsnGS1.2* transgenic tobacco and WT. **(A)** Heights and diameters. **(B)** Leaf lengths and widths. **(C)** Fresh and dry weights. **(D)** Breaking forces. Each error bar represents SD of three biological replicates, Asterisks indicate levels of significance (*t*-test; ^∗^*P* < 0.05, ^∗∗^ *P* < 0.01).

To investigate *PsnGS1.2*’s functions in plant growth and development, the *PsnGS1.2* transgenic lines were generated in tobacco. In total, 22 T2 transgenic lines were generated through the cross-hybridization as described (Wei et al., 2015), and were corroborated to harbor *PsnGS1.2* by genomic PCR. The *PsnGS1.2* expression levels in these transgenic lines were then quantitatively analyzed using RT-PCR. The expression levels of *PsnGS1.2* in several transgenic lines, for instance, L1, L11, L16, and L19, were higher than those in other transgenic lines (**Figure 2D**). Moreover, these *PsnGS1.2* transgenic lines exhibited more vigorous growth during vegetative stage than the WT (**Figures 2E,F**), and thus were chosen for further characterization. However, as shown in **Figure 2F**, the flowering time had no notable difference between the *PsnGS1.2* transgenic lines and the WT (**Figure 2F**). Although there were no significant differences in the diameters and leaf widths (**Figures 3A,B**), the *PsnGS1.2* transgenic lines had increased heights, leaf lengths, fresh and dry weights, and breaking forces by on average 22.4, 20.2, 58.7 and 54.1%, and 23.4% as compared to WT, respectively (**Figures 3A–D**). These results indicated that *PsnGS1.2* overexpression could accelerate vegetative growth and improve biomass for transgenic lines.

### Effects of *PsnGS1.2* Overexpression on Biochemical Levels of Transgenic Tobacco

**FIGURE 4:**
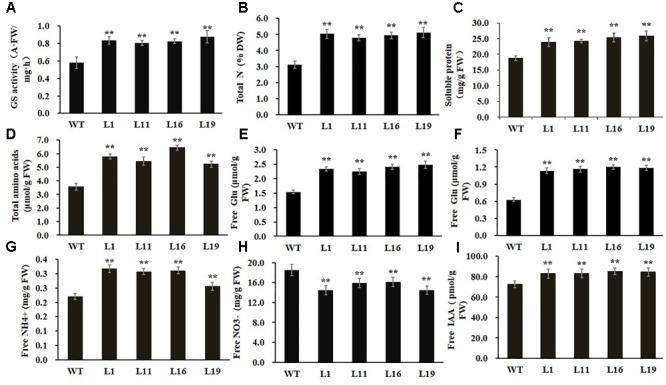
Biochemical levels of *PsnGS1.2* transgenic tobacco and WT. **(A)** Total GS activity. **(B)** Total N content. **(C)** Soluble protein content. **(D)** Total amino acid content. **(E)** Free Glu content. **(F)** Free Gln content. **(G)** Free NH_4_^+^ content. **(H)** Free NO_3_^-^ content. **(I)** Free IAA content. Each error bar represents SD of three biological replicates, Asterisks indicate levels of significance (*t*-test; ^∗^*P* < 0.05, ^∗∗^*P* < 0.01).

To examine whether GS enzyme activity was enhanced in *PsnGS1.2* transgenic tobacco, we examined the GS activities. As shown in **Figure 4A**, the GS activities increased 43.6% in the *PsnGS1.2* transgenic lines as compared to the WT, indicating that the mRNAs of *PsnGS1.2* could be translated into functional proteins. We further analyzed other biochemical contents to investigate whether the N assimilation and metabolism changed accordingly owning to the increased GS activity in *PsnGS1.2* transgenic lines. The results showed that the contents of total N, soluble protein, total amino acid, free Glu, free Gln, and free NH_4_^+^ increased 27.8, 32.1, 43.6, 55.4, 86.6, and 35.7% in the *PsnGS1.2* transgenic lines than those in the WT, respectively (**Figures 4B–G**). In contrast, the free NO_3_^-^ content of *PsnGS1.2* transgenic lines decreased 26.4% as compared with that of WT (**Figure 4H**). Moreover, we also performed GC/MS analysis of free IAA content in tobacco. Compared to WT, the *PsnGS1.2* transgenic lines displayed a 15.6% increase of free IAA content (**Figure 4I**). These results indicated that *PsnGS1.2* overexpression led to significant changes in N assimilation and metabolism, amino acid homeostasis, and also a reinforcement of free IAA biosynthesis in transgenic tobacco.

**FIGURE 5:**
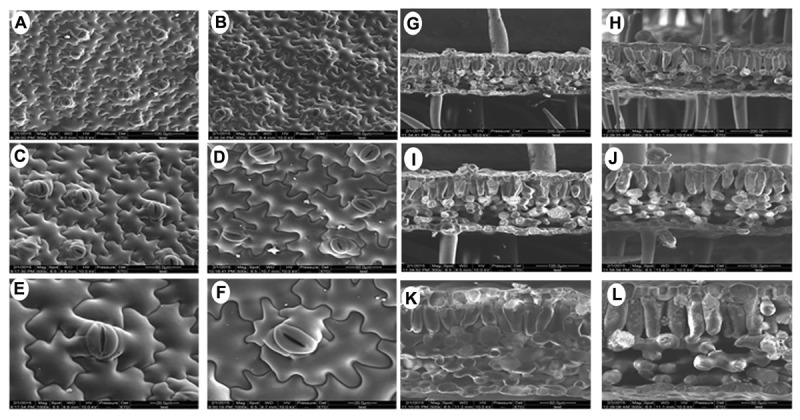
Leaf ultrastructure of *PsnGS1.2* transgenic tobacco and WT. Scanning electron microscope (SEM) of leaf epidermis in WT **(A,C,E)** and *PsnGS1.2* transgenic tobacco **(B,D,F)**. SEM of leaf cross sections in WT **(G,I,K)** and *PsnGS1.2* transgenic tobacco **(H,J,L)**. **(A,B,G,H)**, 300 x magnification; **(C,D,I,J)**, 500x magnification; **(E,F,K,L)**, 1000x magnifications.

**FIGURE 6:**
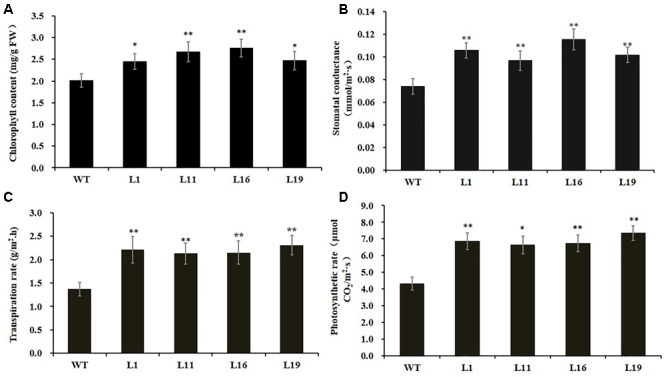
Chlorophyll content and photosynthetic parameters of *PsnGS1.2* transgenic tobacco and WT. **(A)** Content of chlorophyll. **(B)** Stomatal conductance. **(C)** Transpiration rate. **(D)** Photosynthetic rates. Each error bar represents SD of three biological replicates. Asterisks indicate levels of significance (*t*-test; ^∗^*P* < 0.05, ^∗∗^*P* < 0.01).

Since there were obvious differences in the leaf lengths between the *PsnGS1.2* transgenic lines and WT, we suspected the increased leaf lengths were primarily caused by the enlargement of leaf cells. The leaf cell sizes and structures were examined using SEM. The result indicated that the epidermis cells at upper leaf surfaces of *PsnGS1.2* transgenic lines were much larger than those of WT (**Figures 5A–D**), while the stomatal numbers per unit area were decreased about 30% (**Figures 5C,D**), but no changes in the stomatal sizes (**Figures 5E,F**). The anatomical changes in the cross sections of leaves showed that the thickness, volume of palisade and spongy parenchyma in *PsnGS1.2* transgenic lines obviously increased compared to WT (**Figures 5G–L**). Considering that the changes of leaf structures in *PsnGS1.2* transgenic lines might affect the chlorophyll contents and photosynthetic parameters, we measured the chlorophyll content, stomatal conductance, transpiration rate, and photosynthetic rate, which were 29.3, 40.7, 36.4, and 48.6% higher than those of WT, respectively (**Figures 6A–D**). These results suggested *PsnGS1.2* overexpression had influence on leaf structures and photosynthesis of transgenic tobacco.

### Changes of Secondary Cell Walls and Fiber Characteristics in *PsnGS1.2* Transgenic Tobacco

**FIGURE 7:**
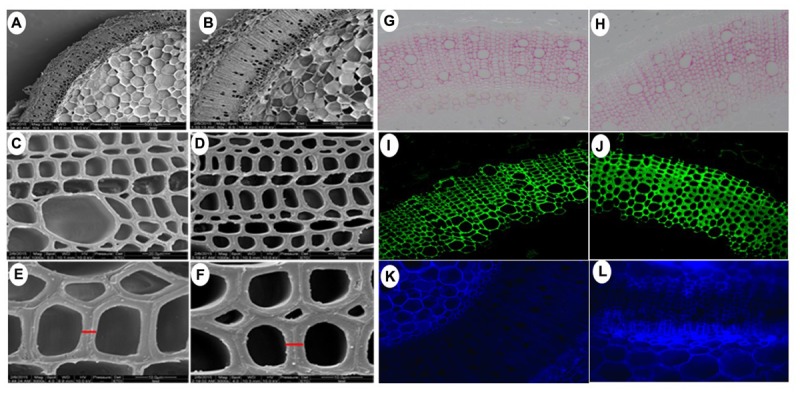
Secondary cell wall thickness and composition in stems of *PsnGS1.2* transgenic tobacco and WT. SEM of cross stem sections of WT **(A,C,E)** and *PsnGS1.2* transgenic lines **(B,D,F)**. **(A,B)**, 1000x magnification; **(C,D)**, 3000x magnification; **(E,F)**, 5000x magnification. Lignin (red color) stained with Phloroglucinol-HCl **(G,H)**, Xylan (green color) stained with LM10 xylan monoclonal antibody **(I,J)**, Cellulose in stem sections stained with Calcofluor White **(K,L)**. **(G,I,K)**, represent WT; **(H,J,L)**, represent *PsnGS1.2* transgenic tobacco.

**FIGURE 8:**
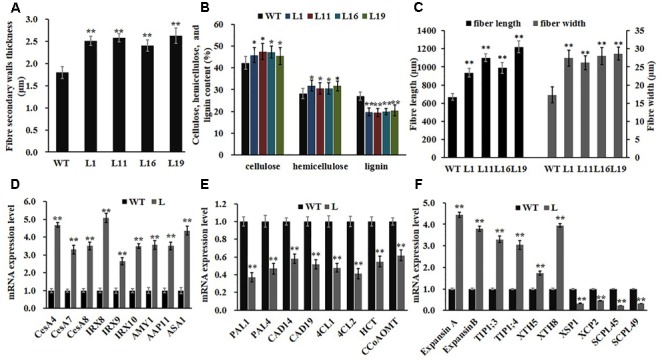
Fiber characteristics and gene expression involved in wood formation and N metabolism in stems of *PsnGS1.2* transgenic tobacco and WT. **(A)** Secondary wall thicknesses. **(B)** Contents of cellulose, hemicellulose, and lignin. **(C)** Fiber lengths and widths. **(D)** Expression levels of genes involved in cellulose (*CESA4, CESA7*, and *CESA8*) and hemicellulose (*IRX8, IRX9*, and *IRX10*) biosynthesis, β-amylase gene (*AMY1*), a membrane transport protein gene (*AAP11*), and a rate-limiting IAA biosynthesis gene (*ASA1*). **(E)** Expression levels of genes involved in lignin biosynthesis (*PAL1, PAL4, CAD14, CAD19, 4CL1, 4CL2, HCT*, and *CCoAOMT1*). **(F)** Expression levels of genes involved in cell expansion (*ExpansinA, ExpansinB, TIP1;3, TIP1;4, XTH5*, and *XTH8*) and programmed cell death (*XSP1, XCP2, SCPL45*, and *SCPL49*). *NtACTIN2* was used as an internal control. The expression of level of each gene in the WT was set to 1, Each error bar represents SD of three biological replicates, Asterisks indicate levels of significance (*t*-test; ^∗^*P* < 0.05, ^∗∗^*P* < 0.01).

Owning to the alternations of growth traits of *PsnGS1.2* transgenic lines, we further examined secondary walls and fiber characteristics in the stems of transgenic lines. Examination of SEM demonstrated that the *PsnGS1.2* transgenic lines exhibited 40.7% thicker secondary walls than those of WT (**Figures 7A–F, 8A**). To identify which component caused augmented secondary cell wall thickening, we used calcofluor and phloroglucinol-HCl to stain cellulose and lignin, respectively, and monoclonal antibody LM10 to label xylan immunologically. The results showed that the lignin content decreased, whereas the deposit of cellulose and hemicellulose increased in the *PsnGS1.2* transgenic lines as compared to the WT (**Figures 7G–L**). Chemical analysis implicated that the contents of cellulose and hemicellulose increased 10.1 and 9.7%, respectively, and the lignin content reduced 23.4% in the *PsnGS1.2* transgenic lines as compared to these in the WT (**Figure 8B**). We also found that the average length and width of fiber in *PsnGS1.2* transgenic lines were about 20 and 18% more than those in the WT, respectively (**Figure 8C**). Taken together, these results implicated that *PsnGS1.2* overexpression had significant influence on secondary walls and fiber characteristics of transgenic tobacco.

### Alternation of Gene Expression in *PsnGS1.2* Transgenic Tobacco

Consistent with changes of secondary walls and fiber characteristics, the expression levels of genes participating in secondary wall principal components biosynthesis, including cellulose (*CesA4, CesA7*, and *CesA8*) and hemicellulose (*FRA8, IRX9*, and *IRX 10*), were significantly up-regulated in *PsnGS1.2* transgenic lines compared to those in WT (**Figure 8D**). In contrast, the expression levels of lignin biosynthetic genes such as *PAL1, PAL4, CAD14, CAD19, 4CL1, 4CL2, HCT*, and *CCoAOMT1* showed significant reduction (**Figure 8E**). Moreover, the genes including *ExpansinA, ExpansinB, TIP1;3, TIP1;4, XTH5*, and *XTH8* involved in the cell expansion and elongation were significantly up-regulated, whereas the genes including *XSP1, XCP2, SCPL45*, and *SCPL49* involved in programmed cell death were notably down-regulated (**Figure 8F**). In addition, the expression levels of *APP11* and *AMY1*, which play important roles in N transportation during xylem formation (Couturier et al., 2010) and starch breakdown (Castro-Rodriguez et al., 2016), respectively, were significantly up-regulated. We also analyzed the expression level of *ASA1*, which catalyzes a rate-limiting step of IAA biosynthesis (Sun et al., 2009), and found its expression level was remarkably increased in the *PsnGS1.2* transgenic lines compared with that in the WT (**Figure 8D**). These results aligned well with the alternations of both N assimilation and metabolism, growth traits, and secondary walls and fiber characteristics observed in the *PsnGS1.2* transgenic tobacco.

## Discussion

As the first enzyme in the N assimilatory pathway, *GS1s* have been proven to play a critical partition role in plant N assimilation and metabolism, growth, and productivity (Fuentes et al., 2001; Habash et al., 2001; Oliveira et al., 2002; Man et al., 2005; Molina-Rueda and Kirby, 2015; Seger et al., 2015; Guan et al., 2016). In this study, a cytoplasmic GS1 type protein *PsnGS1.2*, was isolated from *P. simonii × P. nigra* and characterized by overexpression in tobacco. We observed a number of phenotypic, biochemical, physiological, as well as anatomical changes in the *PsnGS1.2* transgenic tobacco, which resembled the changes that were observed in other *GS1s* transgenic plants (Man et al., 2005; Tabuchi et al., 2007; Zhu et al., 2014; Molina-Rueda and Kirby, 2015). At same time, we also found some distinct functions of *PsnGS1.2*. For example, the free NH_4_^+^ contents in the *PsnGS1.2* transgenic tobacco increased rather decreased, as observed in other studies (Lothier et al., 2011; Molina-Rueda and Kirby, 2015). We speculated that this difference is probably caused by distinct expression pattern of *PsnGS1.2*. The *PsnGS1.2* is predominantly expressed in roots, and may indirectly accelerate NO_3_^-^ reduction to NH_4_^+^ through up-regulation the nitrate transporter genes in roots and nitrate reductase (NR) genes in leaves of *PsnGS1.2* transgenic tobacco, as what was described for *OSGS1;2* (Cai et al., 2009). This led to the decreased NO_3_^-^ content and increased NH_4_^+^ content. In contrast, the *GS1*s that are primarily expressed in phloem tissue of stems may function in the translocation of the N compounds from roots to leaves, and thus their overexpression result in an increase of NH_4_^+^ and NO_3_^-^ contents in the leaves (Lothier et al., 2011; Molina-Rueda and Kirby, 2015). In addition, there are also some *GS1*s, which are constitutively expressed in leaves and play a primary role in the synthesis of Gln using NH_4_^+^ that is converted from NO_3_^-^, and their overexpression causes both NH_4_^+^ and NO_3_^-^ contents decreased in transgenic plants (Martin et al., 2006; Zhu et al., 2014). In addition, it is noteworthy that there are also some *GS1.2* genes that do not have any effects on N assimilation and metabolism, and growth when they are overexpressed (Ortega et al., 2001; Fei et al., 2003).

When *PsnGS1.2* was overexpressed in tobacco, the direct effect was that the N assimilation and metabolic products, such as total N, soluble proteins, total amino acids, free NH_4_^+^, free Glu and Gln contents were significantly altered. Consequently, in order to maintain a balanced metabolism between N and C in response to the increased N assimilation and alternated N compounds, the *PsnGS1.2* transgenic tobacco exhibited a series of phenotypical and physiological changes, such as larger leaf sizes, altered leaf structural features, increased chlorophyll contents, and augmented photosynthesis to improve carbon skeleton assimilation, which resulted in reinforced vegetative growth and biomass production. These results also confirmed previous studies that the augmented N assimilation can accumulate more biomass through promoting photosynthesis that produces more carbon skeletons and leads to the augmented synthesis of starch and sucrose (Nunes-Nesi et al., 2010; Bao et al., 2014).

The *PsnGS1.2* transgenic tobacco contained more cellulose and hemicellulose and reduced lignin, indicating there was a reinforcement of carbon flux to cellulose and hemicellulose instead of lignin biosynthesis pathway. It has been reported that the cellulose contents are positively correlated with vegetative biomass (Novaes et al., 2009), and the increased biomass is concomitant with reduced levels of lignin (Novaes et al., 2010). Thus, the increased contents of cellulose and hemicellulose could contribute directly to the increased biomass in *PsnGS1.2* transgenic tobacco. Moreover, the changes of cellulose and lignin contents did not show any adverse effects on the growth and development for *PsnGS1.2* transgenic tobacco, as they exhibited normal vegetative growths and flowers. As tensile strength is correlated with cellulose content of plant stems (Dhugga, 2007; Voelker et al., 2011), the increase in cellulose of *PsnGS1.2* transgenic tobacco could counteract some mechanical strength reduction due to the decrease of lignin, as indicated in other studies (Ambavaram et al., 2011; Welker et al., 2015). Moreover, we also observed that the *PsnGS1.2* transgenic tobacco displayed longer and wider fibers. On the contrary, in pine *GS1a* transgenic poplar, the fiber lengths increased but the fiber widths did not change significantly (Coleman et al., 2012). It has also been reported that the transcriptome during poplar growth was notably changed in response to high N availability, resulting in alternations of large number of genes in different biological pathways (Cooke et al., 2003; Castro-Rodriguez et al., 2016). As *PsnGS1.2* overexpression caused notable alternation of N assimilation and metabolism, carbohydrate accumulation, and secondary walls and fiber characteristics, we reasoned that the expression of key genes related to these traits should be altered in the *PsnGS1.2* transgenic tobacco. Because the *APP11* encodes an amino acid transporter that plays a critical role in N transportation during the process of xylem differentiation (Couturier et al., 2007), and *AMY1* functions in starch mobilization (Lloyd et al., 2005), the increased expressions of *APP11* and *AMY1* in *PsnGS1.2* transgenic tobacco could account for the alternations of N and C metabolism. Moreover, the genes participating in the secondary wall formation, fiber cell expansion and extension, and programmed cell death exhibited notable alternations in *PsnGS1.2* transgenic tobacco. These results indicated that the *PsnGS1.2* overexpression had an influence on the gene expression of N and C metabolic pathway, as what were observed in previous study (Castro-Rodriguez et al., 2016).

The expression level of *ASA1*, which catalyzes a rate-limiting step of IAA biosynthesis (Sun et al., 2009), was significantly up-regulated in *PsnGS1.2* transgenic tobacco. This is similar to what was observed in Pinus *GS1a* transgenic poplar (Man et al., 2011). Moreover, the *PsnGS1.2* transgenic tobacco exhibited significantly increased contents of Gln which is known to play an important role in enhancing IAA biosynthesis (Mihaljevic et al., 2011). These results suggested *PsnGS1.2* overexpression could promote IAA biosynthesis and lead to increased IAA content in *PsnGS1.2* transgenic tobacco. Considering the roles of IAA in plant growth, development, and wood formation (Moyle et al., 2002; Zhao, 2010), we speculate that the increased IAA content may play a significant role in promoting vegetative growth and secondary wall formation, and fiber characteristics alternation in *PsnGS1.2* transgenic tobacco. However, this hypothesis is still lack of direct biological evidence and need to be further investigated. At the same time, it is also intriguing to study the performance of *PsnGS1.2* transgenic tobacco under low N condition.

## Author Contributions

TL and LL: participated in experiment setup and measurements. MW: participated in experiment measurements. YL: participated in the data analysis. ZQ: participated in editing of the manuscript. CY: revised the manuscript. HW: performed data analysis and wrote manuscript. ZW: designed the experiments, performed data analysis, and wrote manuscript. All the authors read and approved the final version of the manuscript.

## Conflict of Interest Statement

The authors declare that the research was conducted in the absence of any commercial or financial relationships that could be construed as a potential conflict of interest.

## Footnotes

1 http://ncbi.nlm.nih.gov

2 http://www.ebi.ac.uk/Tools/msa/clustalw2/

3 http://www.phytozome.net/

4 http://rsbweb.nih.gov/ij/

5 http://www.graphpad.com/quickcalcs/ttest1.cfm
