# Supplementary material for: The Effect of Poplar PsnGS1.2 Overexpression on Growth, Secondary Cell Wall, and Fiber Characteristics in Tobacco
Source: Front Plant Sci. 2018 Jan 19;9:9. doi: 10.3389/fpls.2018.00009 (PMC5780347; doi:10.3389/fpls.2018.00009)
Supplement: Supplementary file 1 [file Presentation_1.pdf]

Supplementary Material

The Effect of Poplar *PsnGS1.2* Overexpression on Growth, Secondary Cell Wall, and Fibre Characteristics in Tobacco

Tingting Lu[1+], Lulu Liu[1+], Minjing Wei[1], Yingying Liu[1], Zianshang Qu[1], Chuanping Yang[1] Hairong Wei[2*] & Zhigang Wei[1*]

[1]State Key Laboratory of Tree Genetics and Breeding, Northeast Forestry University, Heilongjiang Harbin 150040, P. R. China
[2]School of Forest Resource and Environmental Science, Michigan Technological University, Houghton, MI 49931, USA

*Corresponding authors: Hairong Wei (hairong@mtu.edu) & Zhigang Wei (zhigangwei@nefu.edu.cn)

+ These authors contributed equally to this work

71 *GS1.2* gens from 44 species in the Figure 1B used for polygenetic analysis are listed below:

GusGs1.2-27391348*(Coccomyxa subellipsoidea)*,*MpGS1.2*-27347205*(Micromonas pusilla)*, *MsGS1.2-*27395379*(Micromonas commoda)*,*AlGS1.2a*-16043838*(Arabidopsis lyrata)*, *AtGS1.2a*-19668213*(Arabidopsis thaliana)*,*BstGS1.2a*-30663361*(Boechera stricta)* and *BdGS1.2b*-32813071*(Brachypodium distachyum)*, *BrGS1.2*-30622020 *(Brassica rapa L.)*, *BsGS1.2a*-32865940*(Brassica stricta)*, *CgGS1.2*-28894813*(Calamintha grandiflora)*, *CrGS1.2-2*0894742 *(Capsella rubella)*, *CcGS1.2*-20802556*(Citrus clementina)*, *CrGS1.2*-30784960*(Chlamydomonas reinhardtii)*, *CsGS1.2*-16962156*(Cucumis sativus)*, *EgGS1.2*-32071611*(Eucalyptus grandis)*, *CpGS1.2*-16416662*(Carica papaya)*, *GmGS1.2b*-30494498*(Glycine max)*, *GmGS1.2a*-30486852*(Glycine max)*, *GrGS1.2*-26825532*(Gossypium raimondii)*, *ZmGS1.2a*-31052315*(Zea mays)*, *ZmGS1.2a*-30991251*(Zea mays)*, *MaGS1.2*-32295174*(Musa acuminata)*, *VvGS1.2*-17823766*(Vitis vinifera)*, *KmGS1.2a*-32564201 and *KmGS1.2b*-32566318 *(Kalanchoe marnieriana)*, *OsGS1.2*-33138580*(Oryza sativa)*, *LuGS1.2a*-23156715 and *LuGS1.2b*-23140884 *(Linum usitatissimum)*, *MeGS1.2a*-32360092, *MeGS1.2b*-32332390 and *MeGS1.2c*-32327725*(Manihot esculenta)*, *MMtGS1.2b*-31103933*(Medicago truncatula)*, *FvGS1.2*-27246631*(Fragaria vesca)*, *CsiGS1.2b*-18115172*(Citrus sinensis)*, *PhGS1.2*-32528568*(Panicum hallii)*, *PviGS1.2a*-30251486 and *PviGS1.2b*-30235113 *(Panicum virgatum)*, *StGS1.2a*-24409169*(Solanum tuberosum)*, *PvGS1.2*-27164009*(Phaseolus vulgaris)*, *PtrGS1.2a*-27031680 and *PtrGS1.2b*-27016370*(Populus trichocarpa)*, *PpGS1.2a*-32983393, *PpGS1.2b*-32983472 and *PpGS1.2d*-32916346*(Physcomitrella patens)*, *PpeGS1.2*-32107054*(Prunus persica)*, *SpGS1.2*-31427432*(Salix purpurea)*, *SiGS1.2*-32694972*(Sesamum indicum)*, *SvGS1.2*-32666751*(Setaria viridis)*, *SbGS1.2*-32747754*(Sorghum bicolor)*, *SlyGS1.2a*-27278132, *SlyGS1.2b*-27299078 and *SlyGS1.2c*-27295659*(Solanum lycopersicum)*, *SfGS1.2a*-32612973 and *SfGS1.2b*-32616578*(Sparganium fallax)*, *TcGS1.2*-27460180*(Theobroma cacao)*, *EsGS1.2*-20189742*(Eutrema salsugineum)*, *VcGS1.2*-32884715*(Volvox carteri)*, *LsGS1.2*-1169931*(Lactuca sativa)*, *RsGS1.2*-1526566*(Raphanus sativus)*, *SoGS1.2*-156072358*(Spinacia oleracea)*, *RcGS1.2*-223533499*(Ricinus communis)*, *GhGS1.2*-159138931*(Gossypium hirsutum)*, *PtrGS1.2a*-27016370 and *PtrGS1.2b*-27016370*(Populus trichocarpa)*, *SoGS1.2*-56681313*(Saccharum officinarum)*, *PviGS1.2a*-30251486*(Panicum virgatum)*, *SbGS1.2*-194326198*(Sorghum bicolor)*, *CmGS1.2*-113171384*(Cucumis melo)*, *SmGS1.2*-15408012*(Selaginella moellendorffii)*, *PpGS1.2b*-32983472*(Physcomitrella patens)*, *PpGS1.2c*-32916757*(Physcomitrella patens)*, *PinusGS1a(Pinus sylvestris)*, *PsnGS1.2(Populus simonii ×Populus nigra)*, *PtaGS1.2*-13924490*(Populus tremula × Populus alba)*, *PsGS1.2*-121489632*(Pisum sativum)*.

Table S1 The primer used to amplify the full cDNA of *PsnGS1.2* from *Populous simonii* ×*P. nigra*

Gene Name	Primer	Sequence(5'-3')
PsnGS1.2	PsnGS1.2cDNAF	ATGTCTCTCCTTAATGATCTCATC
	PsnGS1.2cDNAR	CTATGGCTTCCAGATAATGGTAG

Table S2 The primer used for amplify the full cDNA of *PsnGS1.2* without the termination codon

Gene Name	Primer	Sequence(5'-3')
PsnGS1.2	PsnGS1.2subF	TCTAGACTGGTACCCATGTCTCTCCTTAATGATC
	PsnGS1.2subR	CTAGTCAGTCGACCCTGGCTTCCAGATAATGGTAG

Table S3 The primers used in the *PsnGS1.2* overexpression transgenic tobacco (*Nicotiana tabacum*)

Gene Name	Primer	Sequence(5'-3')
PsnGS1.2	PsnGS1.2overexF	TGCTCTAGAATGTCTCTCCTTAATGATCTCATC
	PsnGS1.2overexR	CGGGGTACCCTATGGCTTCCAGATAATGGTAG
	PROKIIF	AAGACCGCAACAGGATTC
	PROKIIR	CGCACAATCCCACTATCCTT

Note: The underline represents an enzyme digestion site.

Table S4 The primers used in quantitative RT-PCR analysis of *Populous simonii* ×*P. nigra* and *PsnGS1.2* transgenic lines

Gene Name	Primer	Sequence(5'-3')
PsnGS1.2	PsnGS1.2RTF	ATCGAGAAGCTTGGTCTGCGC
	PsnGS1.2RTR	TCAGCTATCATTGATGTGACAACA
PsnActin1	Actin1F	AGGCAGGTTTCGCAGGAGATGA
	Actin1R	TCCATCACCAGAATCCAGCACA
NtActin2	Actin2F	ATCGCCATTGTTCTTTC
	Actin2R	TGTCTCCGTTCTTCACTTC

Table S5 The primers used to analyze expression levels of genes involved in fibre development, IAA biosynthesis, membrane transport protein, β-amylase, and secondary wall formation biosynthesis of *PsnGS1.2* lines

Gene Name	Accession number	Primer	Sequence(5'-3')
NtCesA4	JN009108.1	NtCesA4RTF	TATGGATACGGGACTGTTGCATGG
		NtCesA4RTR	TTACCACCACCTTTACTGCCTCCG
NtCesA7	JQ735443.1	NtCesA7RTF	TTGCTTCGTCCAGTTTCCTCAAAG
		NtCesA7RTR	CTGGACCTTGTATTCCGTCTAGGC
NtCesA8	JQ735445.1	NtCesA8RTF	CCAAAGCTGCAGATGATGGAGAGT
		NtCesA8RTR	TTGAGTGCATCAGAGAAACCAGCA
NtIRX8	JQ735447.1	NtIRX8RTF	TGAGCATTCTACCAATTCCAATGC

		NtIRX8RTR	CTGCACGAGTGATGAAGCAACAAC
NtIRX9	JQ735449.1	NtIRX9RTF	ACATGGCCAATGGCATTATTATCT
		NtIRX9RTR	CACTTGAAACTCTAATTGGAGGCC
NtIRX10	JQ735450.1	NtIRX10RTF	CCCTGTTTACACCACTTGTGACCT
		NtIRX10RTR	AGAAATGATCAGCTCCCTCTGTCC
NtPAL1	M84466.1	NtPAL1RTF	ATTGGAGCTTTCGAAGATGAATTG
		NtPAL1RTR	CTGTTCCAAGCTCCTTTCTCACAA
NtPAL4	X78269.1	NtPAL4RTF	CTGCAATTGCCAACAGGATAAAGG
		NtPAL4RTR	TGTCCATTGCACATTGCTGTGAAC
NtCAD14	X62343.1	NtCAD14RTF	CTGTTGGCCATCCTCTTGAACCTT
		NtCAD14RTR	CTTCCTGTGATGCTCTTTCTCCCG
NtCAD19	X62344.1	NtCAD19RTF	TGATACTGTCCCTGTTGGCCATCC
		NtCAD19RTR	TTCCAGTGATGCTCTTTCTCCCGA
Nt4CL1	U50845.1	Nt4CL1RTF	ATGGTTACACACTGGCGACATTGG
		Nt4CL1RTR	ACAGCAGCATCAGAAATGTTGGGA
Nt4CL2	U50846.1	Nt4CL2RTF	TGAGCTCGAAGCTCTCCTTCTCAA
		Nt4CL2RTR	CTTCAGTAATGGTGGATCCGTTGG
NtHCT	AJ507825.1	NtHCTRTF	ACTCCTATTGCAGTCGCAGGTGAT
		NtHCTRTR	ACGTATGTGCACCACGAACAAGAG
NtCCoAOMT	U38612.1	NtCCoAOMTRTF	CCTGAGCCCATGAAAGAGCTAAGA
		NtCCoAOMTRTR	CAATCTCCATTGTGTTCTTGGCAT
Nt4CL3	XM_009605419.1	Nt4CL3RTF	TAGTGTTGAAGCCCAGAAAGC
		Nt4CL3RTR	TGTCCACTGCTGCCTGCTAT
ExpansinA	XM_009627111	NtExpasionARTF	ACACAGCAGCATTGAGTACAGC
		NtExpasionARTR	GGCATAGTTTGGTGGGCAG
ExpansinB	XM_016579143	NtExpassionBRTF	TTCACCAACCCGTGCTTCAG
		NtExpassionBRTR	CCACAAGATCCTCCGTCGCT
TIP1;3	XM_009782262	NtTIP13TF	GCTGGATTGATTTCGGCG
		NtTIP13TR	CCTCCAGAAATGTTTGCTCCTAC
TIP1;4	XM_009782262	NtTIP14TF	TGCCTTTGCCCTTTTTGTG
		NtTIP14TR	GGCAATCCAATACAAAACACTCC
XTH5	XM_009613022	NtXTH5RTF	GTTCTTGACAGGTCTTCAGGAGC
		NtXTH5RTR	AAATGCAGTAACAACACCAGCG
XTH8	XM_009802222	NtXTH8TF	AAAGCAGGATGTGGATTTATGACT
		NtXTH8TR	TCATCTCTTGTTGGTCCTGCTC
XSP1	XM_009764284	NtXSP1RTF	CTTCTGCTGGTTTCTGTCTTATCA
		NtXSP1RTR	AAATAGCGATTCTTCGTTCACC
XCP2	XM_009798142	NtXCP2RTF	TACTCTTCAGATGACTTGACTTGC
		NtXCP2RTR	CGTTCAGTTCAGTAGACTTCTCAT
SCPL45	XM_009600823	NtSCPL45RTF	AGCCTTTGGTTTTGTGGTTG
		NtSCPL45RTR	ATTTGCCTCTTTGTTCCAGC
SCPL49	XM_009786981	NtSCPL49RTF	CATCCCTCTTTCTTACTCTGCTTC
		NtSCPL49RTR	TGGGAATTTGGGAGTAGAAGATAG
ASA1	XM_009763313	NtASA1TF	TACCATTTTTCCAAGAACCCAAC
		NtASA1TR	AAGGGGTTTTTCTTGCTAGTGTT
AAP11	XM_009795282	NtAAP11TF	ATAACACAAACACACACACACGC

		NtAAP11TR	AAAGTTTTTTTGAACATCTCCTCT
AMY1	XM_009760233	NtAMY1TF	TGGGAATCAAGTAATCAGCAAGG
		NtAMY1TR	AACCAAACATGAGTAATTCCGGC
